# Shift in beneficial interactions during crop evolution

**DOI:** 10.1111/eva.13390

**Published:** 2022-05-19

**Authors:** Hélène Fréville, Germain Montazeaud, Emma Forst, Jacques David, Roberto Papa, Maud I. Tenaillon

**Affiliations:** ^1^ AGAP Univ Montpellier CIRAD INRAE Institut Agro Montpellier France; ^2^ 27213 Department of Ecology and Evolution University of Lausanne Lausanne Switzerland; ^3^ 9294 Department of Agricultural, Food and Environmental Sciences Università Politecnica delle Marche Ancona Italy; ^4^ Génétique Quantitative et Evolution – Le Moulon INRAE, CNRS, AgroParisTech Université Paris‐Saclay Gif‐sur‐Yvette France

**Keywords:** agroecology, biotic interactions, facilitation, kin selection, niche complementarity, plant breeding

## Abstract

Plant domestication can be viewed as a form of co‐evolved interspecific mutualism between humans and crops for the benefit of the two partners. Here, we ask how this plant–human mutualism has, in turn, impacted beneficial interactions within crop species, between crop species, and between crops and their associated microbial partners. We focus on beneficial interactions resulting from three main mechanisms that can be promoted by manipulating genetic diversity in agrosystems: niche partitioning, facilitation, and kin selection. We show that a combination of factors has impacted either directly or indirectly plant–plant interactions during domestication and breeding, with a trend toward reduced benefits arising from niche partitioning and facilitation. Such factors include marked decrease of molecular and functional diversity of crops and other organisms present in the agroecosystem, mass selection, and increased use of chemical inputs. For example, the latter has likely contributed to the relaxation of selection pressures on nutrient‐mobilizing traits such as those associated to root exudation and plant nutrient exchanges via microbial partners. In contrast, we show that beneficial interactions arising from kin selection have likely been promoted since the advent of modern breeding. We highlight several issues that need further investigation such as whether crop phenotypic plasticity has evolved and could trigger beneficial interactions in crops, and whether human‐mediated selection has impacted cooperation via kin recognition. Finally, we discuss how plant breeding and agricultural practices can help promoting beneficial interactions within and between species in the context of agroecology where the mobilization of diversity and complexity of crop interactions is viewed as a keystone of agroecosystem sustainability.

## 1 INTRODUCTION

In natural ecosystems, plants interact with their physical and chemical environment (e.g., temperature, water, light, day length, atmospheric CO_2_, nutrients, soil acidity, soil texture), which effects on ecosystem composition have been thoroughly studied (Begon et al., 2005). For instance, widespread changes in phenology have been documented in many plant populations as a result of climate change (Franks et al., 2014). In addition, plants are part of a rich network of interacting organisms. Such biotic interactions include intraspecific interactions as well as interspecific interactions (with e.g., pollinators, pests, microorganisms, and other plant species), either positive or negative, and are important driving forces in shaping plant communities (Bardgett & Wardle, 2010; Begon et al., 2005).

Agricultural practices have reduced the complexity of crop abiotic and biotic interactions, a trend exacerbated since the Green Revolution. Before the advent of modern breeding, agricultural settings were made of multispecies multigenotype combinations (Harlan, 1992). An emblematic example is the milpa farming system, where maize is intercropped with common beans (*Phaseolus* spp.) and squashes (*Cucurbita* spp.), three‐sister species forming the backbone of pre‐Columbian agriculture (Lopez‐Ridaura et al., 2021). Modern agriculture has highly reduced the network of interactions within and between plant species by (i) progressively abandoning intercropping and crop rotation, (ii) removing weeds from the field, and (iii) eroding genetic diversity within crops with the adoption of monogenotypic varieties grown in pure stands. Intensive agriculture has further diminished abiotic and biotic interactions through the standardization of environments at the expense of fossil energy. For example, practices such as ploughing and fertilization have contributed to buffer physical and chemical heterogeneities of the environment. In addition, chemical protection against weeds, insects, and microorganisms have excluded targeted species, and most likely other untargeted species, from the network of species interacting with crops.

In this context, agroecology aims at designing agroecosystems that benefit from abiotic and biotic interactions in place of fossil energy and chemical inputs (Altieri, 1989). To do so, we need to better understand how domestication and breeding have shaped crop interactions with their abiotic and biotic environment, and have potentially eroded useful genetic variation. The loss of genetic variation associated with the ability of crops to interact with abiotic factors has been documented for many traits, including the loss of seed dormancy (Wang et al., 2018) and the loss of responsiveness to photoperiod (Cortinovis et al., 2020) and to vernalization (Comadran et al., 2012; Iqbal et al., 2020; Yan et al., 2004). For instance, while seed dormancy allows nonsimultaneous seed germination in natural environments, it has been highly counter‐selected by humans to facilitate crop management and harvest as shown in rice, tomato, and soybean (Wang et al., 2018). Yet, how domestication and breeding have affected crop interactions with biotic factors is much less documented.

**FIGURE 1:**
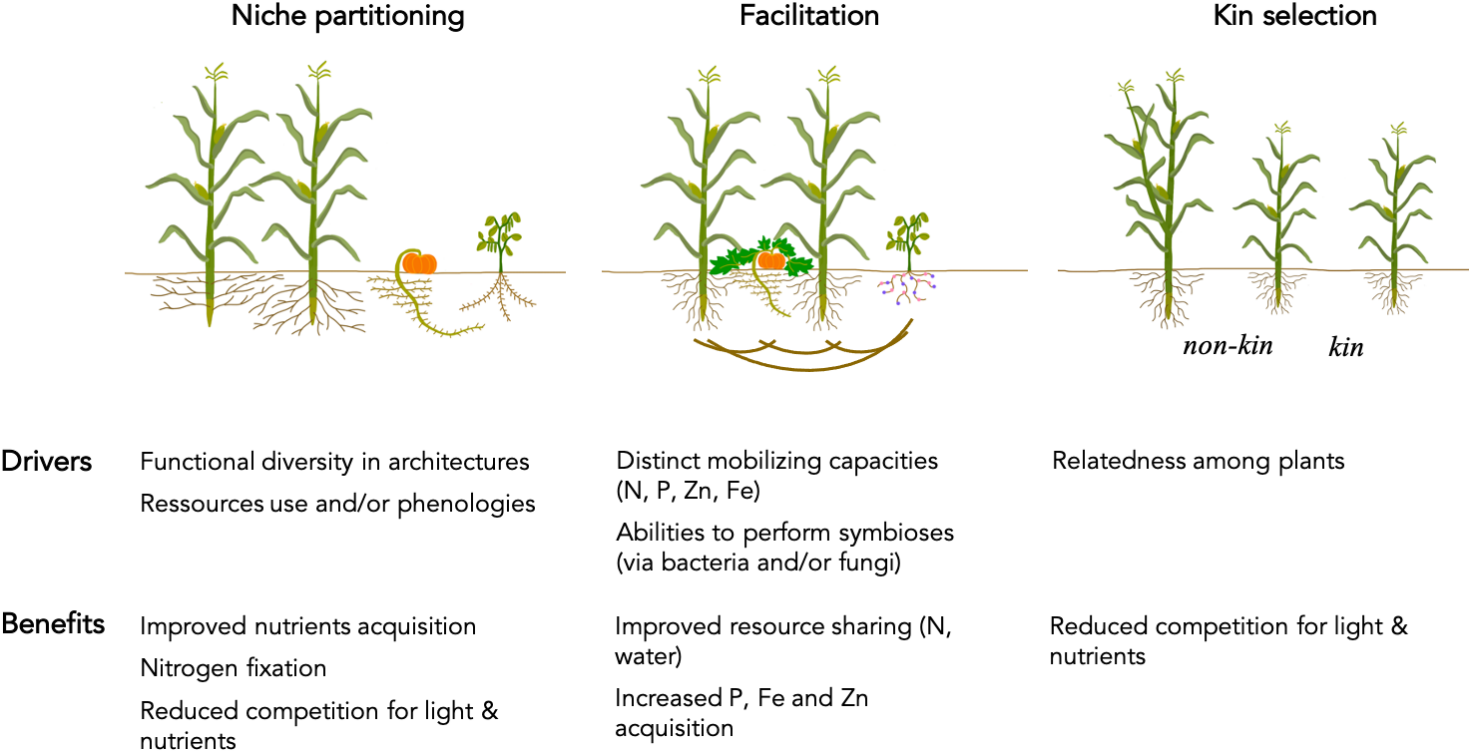
Illustration of the three main mechanisms resulting in crop beneficial interactions: niche partitioning, facilitation, and kin selection. Prototypes of plants are illustrated with non‐N fixing species represented by maize and squash, and N‐fixing species represented by bean. For the sake of clarity, only complementarity of root architecture is illustrated for niche partitioning; as for facilitation we are illustrating bean nodules responsible for N‐fixation as well as conservation of soil moisture by squash leaves; kin selection relies on the relatedness among interacting plants

Among biotic interactions, beneficial interactions result from mechanisms acting both at the intraspecific and the interspecific levels such as niche partitioning (Macarthur & Levins, 1967) and facilitation (Callaway et al., 2002). Niche partitioning (Figure 1) concerns spatial complementarity of canopy and roots—that maximize exploitation of light and soil resources (Brooker et al., 2015), temporal complementarity (Yu et al., 2015), as well as complementarity in resource types (Bedoussac et al., 2015). Facilitation (Figure 1) is achieved when a genotype alters features of the local environment to the benefit of neighboring genotypes of the same or different species (Callaway et al., 2002), and includes positive interactions between plants and microorganisms. Beneficial interactions arising from niche partitioning and facilitation are fueled by genetic and functional diversity. Beneficial interactions can also result from kin selection (Figure 1), a mechanism acting exclusively at the intraspecific level (Hamilton, 1964). Kin selection refers to the evolutionary process by which selection favors a cooperative phenotype because of the fitness benefit it provides to a genetically related interacting individual (Hamilton, 1964; West et al., 2007). Compared with niche partitioning and facilitation, kin selection is thus promoted by genetic similarity among interacting individuals (Biernaskie, 2022; Hamilton, 1964; Montazeaud, Rousset, et al., 2020). In addition, while kin selection occurs within species, niche partitioning and facilitation can result from natural selection leading to beneficial interactions both within or among species (Meilhac et al., 2020; Zuppinger‐Dingley et al., 2014). Beneficial interactions can also emerge incidentally by assembling a diverse set of phenotypes selected in different contexts; this is nowadays the rationale when growing species or genotype mixtures in agriculture. Note that the relative role of incidental by‐products benefits and benefits driven by natural selection can be hard to distinguish.

In contrast to negative biotic interactions involving pathogens, herbivores, and weeds, beneficial interactions within and among species have received much less attention in crops. Yet, they are particularly relevant to face the need of developing more sustainable agriculture (Altieri, 1989; Barot et al., 2017). In this review, we focus on beneficial interactions resulting from the three main mechanisms cited above, that is, niche partitioning, facilitation, and kin selection, which are all potentially relevant for improving agriculture sustainability (Figure 1). We first review the impact of domestication and subsequent breeding on beneficial interactions within and between species. We then build on these historical patterns to discuss how they could be used as agroecological levers and targeted in future breeding programs.

## 2 BENEFICIAL INTERACTIONS ARISING FROM NICHE PARTITIONING

In crowded environments, plants compete for light, water, and nutrients. The niche partitioning theory states that communities made of organisms which differ in their ecological niches are more productive than communities in which organisms have similar niches, notably because competition is reduced when individuals differ in their resource requirements and use (Macarthur & Levins, 1967). Initially developed in ecology to explain species co‐existence in natural ecosystems, this theory was further used for explaining the positive relationship often observed between species richness and ecosystem functioning (Chase & Leibold, 2003), such as increased productivity (Loreau & Hector, 2001) and increased temporal stability (Tilman, 1999) in species‐rich ecosystems. In agronomy, niche partitioning has been proposed as a central mechanism to explain overyielding, that is, increased productivity in intercropping systems where multiple species are grown in association compared to the productivity of their respective components grown alone (Fukai & Trenbath, 1993).

Niche partitioning can result from differences in the architecture of aerial parts and/or root systems (spatial complementarity), from differences in plant development such as phenology and therefore timing in resource use (temporal complementarity), and from differences in plant resource needs (for instance, NO_3_^−^‐N *versus* NH4^+^‐N). Spatial complementarity has been described in trees where canopy stratification—significant height difference—in mixtures of *Eucalyptus globulus* and *Acacia mearnsii* reduces competition for light in comparison with monocultures, as shown by an increase of diameter growth and above‐ground biomass in mixtures compared to sole‐stands (Forrester et al., 2004). In annual plants, simulations of nutrient uptake and resource use in assemblages of the three‐sister species constituting the milpa (maize‐bean‐squash) indicate that root architecture complementarity results in increased biomass production on N‐deficient soils (Postma & Lynch, 2012). Differences in root architecture among these crops reflect a diversity of nutrient foraging strategies, with shallow, more vertical, and deep soil exploration soil for maize, bean, and squash, respectively. These differences translate into a more equal root distribution within soil (Zhang et al., 2014), corroborating results found for maize–wheat intercropping (Li et al., 2006).

At the intra‐specific level, agroecology has stimulated many calls to benefit from complementarity between genotypes (Barot et al., 2017; Hajjar et al., 2008; Litrico & Violle, 2015). It has been shown that variety mixtures, *that is*, the simultaneous cultivation of multiple genotypes of the same species within fields, have a small yield advantage over mono‐genotypic stands, with overyielding ranging from +2% to +5% (Borg et al., 2018; Kiaer et al., 2009; Reiss & Drinkwater, 2018; Smithson & Lenne, 1996). Yet, while this result is consistent with niche partitioning, experimental evidence testing for this mechanism are still lacking. For example, niche partitioning has been shown to play a limited role in the overyielding achieved by rice varietal mixtures in Chinese traditional systems (Revilla‐Molina et al., 2009). At a lower‐scale, mixtures of near‐isogenic lines of rice chosen to only differ in their root depth showed no yield advantage over mono‐genotypic stands (Montazeaud et al., 2018).

**FIGURE 2:**
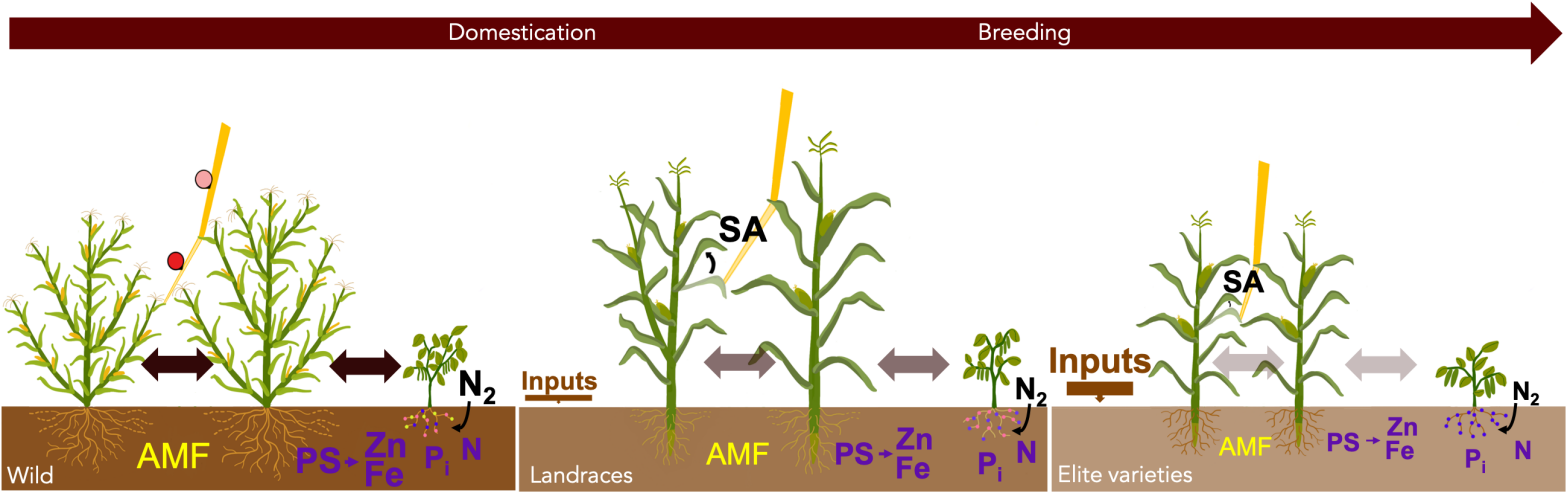
Impact of domestication and breeding on intra‐ and interspecific biotic interactions. Prototypes of wild plants/traditional landraces/elite varieties are illustrated. A non‐N fixing species, here represented by maize, is represented in monospecific stand (left of each panel) and in mixture with a N‐fixing species, here represented by bean. Note that in the former, distance between plants is reduced in landraces and even further reduced in elite varieties. Evolution toward modern crops include an overall reduction of phenotypic (and genetic) diversity, a reduction of tillering/branching, and selection for larger edible parts (ears, pods, and grains) as well as modification of root architecture. In addition, there is a trend toward plasticity reduction in roots (illustrated by dashed roots in wild forms) and in aerial parts (illustrated here with leaf orientation in response to shade avoidance = SA). Loss of genetic diversity and plasticity have likely both contributed to reduce potential complementarity within‐ and between species. Belowground, impoverishment of the soil compensated by increased inputs is accompanied by a reduction of AMF and atmospheric nitrogen (N_2_) fixation by rhizobia in root nodules, with a decrease in the diversity of rhizobia strains colonizing nodules (as shown by the colored points on the soybean root). In addition, facilitation occurring via secretion of root exudates is impacted as illustrated here with a reduced production of phytosiderophores (an in turn of iron and zinc) and availability of inorganic phosphorus (Pi)

The strong loss of genetic and phenotypic diversity that crops experienced during domestication and breeding (Glemin & Bataillon, 2009; Milla et al., 2018) has undoubtedly impacted the potential for niche complementarity to be mobilized from modern genetic pools (Figure 2). At the interspecific level, in agreement with this idea, overyielding is stronger in species mixtures made of crop ancestors than in mixtures made of their domesticated counterparts, likely as an effect of stronger aboveground trait variation in the ancestors (Chacon‐Labella et al., 2019). Moreover, it has been shown that trait differences increase over time when species evolve in mixture, which enhance complementarity effects compared to communities made of species that evolved in monoculture (Meilhac et al., 2020; Zuppinger‐Dingley et al., 2014). In elite germplasm, it is very unlikely that such trait divergence evolved in the recent history of modern breeding since most species have been selected and cultivated in monospecific and monogenotypic stands. Reduced potential for niche complementarity also holds true at the intraspecific level. For instance, compared with organic varieties and landraces, modern wheat varieties display a reduced capacity of ammonium (NH_4_^+^) uptake. Such specialization reduces the opportunities for complementarity between varieties using different forms of nitrogen (Cantarel et al., 2021). Evaluation of eleven functional traits in modern and ancient wheat varieties revealed an average level of variability among varieties <30% of that observed among wild Pooideae species. Although reduced compared to wild progenitors, remaining variation for those traits among wheat varieties is potentially useful for exploitation of functional complementarity in mixtures (Cantarel et al., 2021; Montazeaud, Violle, et al., 2020). High variation was also found in wild emmer and emmer compared to durum wheat variety for shoot and root traits (Gioia et al., 2015).

Complementarity can arise from phenotypic plasticity when co‐occurrence of species and genotypes promote trait differentiation that leads to stable coexistence (Turcotte & Levine, 2016). At the interspecific level, such pattern has been shown within grassland communities as a result of plasticity in plant height (Meilhac et al., 2020). Phenotypic plasticity has also been shown to enhance light acquisition in mixed stands of wheat and maize (Zhu et al., 2015, 2016). At the intraspecific level, a convincing example comes from sunflower where shifts in stem inclinations at high density stand increase spatial complementarity for light and lead to increased oil yield per unit area (Lopez Pereira et al., 2017). Such response is part of the shade‐avoidance syndrome describing phenotypic plasticity on morphological and physiological traits involved in competitive interactions in response to a change in light quantity and quality (Schmitt & Wulff, 1993). Few studies suggest that phenotypic plasticity triggering stronger niche complementarity might have been counter‐selected during crop evolution (Figure 2). For instance, the teosinte allele of the *TB1* gene introgressed in a modern maize background confers greater phenotypic plasticity and responsiveness to light than the maize allele (Lukens & Doebley, 1999). In wheat, recent results suggest that Green Revolution genes introduced in the 1960s to improve fertilizer responsiveness might also have reduced plant height plasticity (Colombo et al., 2022). Similarly, in barley and wheat, wild versus domesticated forms, and landraces versus modern cultivars, display greater plasticity in root traits in response to heterogeneous nutrient availability (Grossman & Rice, 2012). Yet, how phenotypic plasticity on traits involved in plant–plant interactions has evolved during crop domestication and breeding and how plastic responses promote beneficial interactions still deserve much more investigation.

## 3 BENEFICIAL INTERACTIONS ARISING FROM FACILITATION

Facilitative interactions describe the effect of a species on its local environment in such a way that it improves the growth and development of other species (Callaway, 1995). Facilitation can be direct, for example, through modifications of the physicochemical environment (increased temperature, enhanced soil moisture), or indirect, for example, through the attraction of mutualistic species such as soil bacteria or mycorrhizal fungi (Brooker et al., 2008).

Beneficial interactions through direct facilitation can occur in crops when one component of the mixture provides a physical support to the others, reducing the risk of lodging. This has been demonstrated in variety of mixtures in barley (Creissen et al., 2016) and rice (Revilla‐Molina et al., 2009), and in species mixtures, for instance in the milpa system where maize provides a support to climbing bean (Lopez‐Ridaura et al., 2021). Direct facilitation can also be achieved when one species increases the phytoavailability of water, nutrients (e.g., P or N), or micronutrients for other species (Li et al., 2014; Shen et al., 2005; White, George, Dupuy, et al., 2013; White, George, Gregory, et al., 2013; Zhang et al., 2010), or when one genotype increases the availability of resources for other genotypes at the intraspecific level (Barot et al., 2017; Brooker et al., 2016; Hajjar et al., 2008). This pattern has been documented in crops for a wide range of resources (Figure 2). Facilitation for water availability can be illustrated by the example of “hydraulic lift,” a process by which some species increase soil moisture on the upper soil layers by releasing water from their own roots (Caldwell et al., 1998; Prieto et al., 2017). Increase water use efficiency in the Milpa (Figure 1) results from conservation of soil moisture by squash leaves covering the soil surface (Zhang et al., 2014). Likewise, facilitation for phosphorus (P) availability occurs in many crop species assemblages. In intercropping agrosystems of maize and faba bean, the uptake of phosphorus (P) by the bean results in rhizosphere soil acidification through the production of root exudates, which in turn enhances inorganic P availability in the soil and facilitates subsequent uptake by maize (Li et al., 2014). In P‐deficient soil, overyielding of intercropped maize and faba bean reached 26% and 43% compared with monocropped faba bean and maize, respectively, part of it being attributable to belowground interactions (Li et al., 2007; Zhang, Zhang, et al., 2016; Zhang, Liu, et al., 2016). A similar pattern has been documented in associations of chickpea/maize (Li et al., 2004), lupin/wheat (Cu et al., 2005), common bean/wheat (Li et al., 2008), and faba bean/wheat (Li et al., 2016). Similarly, Fe or Zn can hardly be assimilated by some species, which then benefit from the presence of phytosiderophore‐producing species able to extract these micronutrients from soil organic complexes (Li et al., 2014). For example, graminoid species secrete more phytosiderophores that bind Fe^3+^ in the rhizosphere when there is Fe deficiency, enhancing Fe availability for other intercropped species (Dai et al., 2019).

Indirect evidence suggests that human selection might have shaped belowground facilitative interactions among plants (Figure 2). For instance, phytosiderophore exudation has been shown to be around four times higher in wild *Aegilops* accessions than in *Triticum aestivum* cultivars (Neelam et al., 2010), and the chemical nature of root exudates has changed between wild and domesticated tetraploid wheat species (Iannucci et al., 2017). This might come from relaxed selection on nutrient‐mobilizing traits due to strong shifts in soil conditions associated with sedentarization and increased nutrient inputs.

## 4 BENEFICIAL INTERACTIONS ARISING FROM MICROORGANISMS‐TRIGGERED FACILITATION

Many crops benefit from positive interactions with soil microbes that can indirectly trigger synergies within and among plant species. For instance, legumes benefit from the capacity of fixing atmospheric N through symbiosis with a group of soil bacteria collectively called rhizobia. The association of non‐N‐fixing crops with legumes triggers facilitation processes resulting in increased N availability for the former via rhizobial N fixation in the latter. For instance, root exudates from maize promotes faba bean nodulation and N_2_ fixation (Li et al., 2016), hence increasing N availability for maize. Likewise, wheat intercropped with faba bean increases nodulation compared with monocropped faba bean (Liu et al., 2017). Positive interactions with micro‐organisms also include fungi, the most prevalent mutualistic partners in plants, including obligate biotrophic arbuscular mycorrhizal fungi (AMF). Such fungi rely on carbon provided by their hosts and furnish nutrients such as N and P—following solubilizing of mineral forms as well as biotransformation of organic compounds—and can also provide better resistance to stress to their hosts (Berruti et al., 2016). There is now evidence that the stability of this mutualism is based on a reciprocal regulation of carbon exchanges so that the reward of the most beneficial partners among different mycorrhizal strains enforces cooperation between the host plant and its associated AMF (Kiers et al., 2011; but see Walder & Heijden, 2015). Interestingly, the presence of AMF can increase below‐ground complementarities between varieties as shown in maize, where mycorrhizal mixtures showed overyielding and nonmycorrhizal mixtures did not (Wang et al., 2020). Note that domesticated plants exhibit contrasted capacity to benefit from the common mycorrhizal network (CMN). For example, in flax/sorghum association, sorghum invests twice as much as flax into the CMN but gets little in return (Walder et al., 2012). In contrast, in rice/mung bean association, intercropping improves AM fungal colonization of roots with shared benefits for the two crops in P and N uptake, and N transfer from the bean to the rice (Li et al., 2009).

Responsiveness of crops to their micro‐organism partners may have been affected by agronomic practices and/or selection on domesticated traits (Figure 2). Indeed, plant growth and health heavily rely on fertilizers and pesticides in modern agrosystems, such that selective pressures for plant‐microbial exchanges of nutrients might have been relaxed (Philippot et al., 2013). Moreover, human‐mediated selection has affected many traits such as root morphology and physiology (e.g., exudates) that are essential components of plant‐microbial symbioses (Sawers et al., 2018). Several studies suggest a negative impact of human selection on the diversity of rhizospheric microbial communities (Brisson et al., 2019; Mutch & Young, 2004; Spor et al., 2020) but, more importantly, on the strength of beneficial interactions with bacteria. For instance, it has been shown that old cultivars of soybean have a higher yield response to symbiosis and a higher proportion of effective strains in their nodules than recent ones when inoculated with a mixture of effective and ineffective N_2_‐fixing bacteria (Kiers et al., 2007). Likewise, modern wheat cultivars are less capable than ancient cultivars of interacting with multiple strains of a growth‐promoting rhizobacterium that enhances plant growth under water‐stress conditions and nutrient deprivation (Valente et al., 2020).

Studies documenting plant interactions with fungi indicate that domestication has impacted more strongly the composition of fungal communities than bacterial ones (Leff et al., 2017). A comparison of wild and domesticated forms in 14 crops indicates that wild relatives engage in mutualistic interactions with mycorrhizal fungi irrespective of P availability, while P‐fertilization reduces mycorrhizal engagement in domesticated forms (Martin‐Robles et al., 2018). Compared with modern cultivars, older wheat cultivars display enhanced benefit from fungal symbiosis translating into greater plant growth and dry weight (Hetrick et al., 1992). Note that modern cultivars sometimes display enhanced colonization as in maize (An et al., 2010; Sangabriel‐Conde et al., 2014), oat (Koide et al., 1988) and tomato (Bryla & Koide, 1990)—but have evolved a loss of dependence/responsiveness to AMF (Zhu et al., 2001). They indeed capture P more efficiently directly from the soil than do older cultivars.

## 5 BENEFICIAL INTERACTIONS ARISING FROM KIN SELECTION

Increase in frequency over generations of competitive phenotypes is a frequent outcome of natural selection within populations. Yet, groups of competitive phenotypes of the same species may have a low productivity due to stronger competitive interactions among plants and higher investment in resource harvesting at the expense of seed production. Such a negative correlation between individual competitiveness and seed production of the group is a classic prediction of evolutionary game theory (see Anten and Vermeulen (2016) for a review, and Cabal et al. (2020) for a recent example). It has been empirically well documented by agronomists for plant height (Jennings & Dejesus, 1968; Khalifa & Qualset, 1974; Suneson, 1949; Suneson & Wiebe, 1942): tall plants invest extra in acquiring light resources at the expense of short plants, lowering field grain production (Falster & Westoby, 2003). Promoting cooperation among plants by targeting weak competitor phenotypes has thus been around since the Green Revolution (Donald, 1968). The evolution of cooperative phenotypes is at the core of the kin selection (KS) theory, which aims at understanding the evolution of social traits in response to intraspecific interactions (Hamilton, 1964). The KS theory predicts that a cooperative phenotype can be favored by kin selection if the performance of conspecific individuals is sufficiently increased by the focal individual's phenotype, and if these “recipient” individuals are genetically related to the focal individual. Multiple theoretical studies have already discussed the relevance and applicability of KS principles in plant breeding (Biernaskie, 2022; Montazeaud, Rousset, et al., 2020). In the following, we discuss whether human‐mediated selection may have acted as KS to promote cooperation in crops either through unconscious or deliberate selection.

The very few studies investigating phenotypic variation on plant height along a domestication gradient suggest that tall competitive phenotypes have first increased in frequency in emerging crop species (Figure 2). Such temporal pattern was documented in maize, barley, sunflower (Milla et al., 2014), and durum wheat (Roucou et al., 2018). Recent theoretical work suggests that mass selection has reinforced this pattern (Montazeaud, Rousset, et al., 2020; Murphy, Swanton, et al., 2017; Murphy, Van Acker, et al., 2017), thereby contributing to the loss of interesting phenotypes for promoting cooperation within species. During the evolutionary history of seed crops, farmers have not only selected within field by picking plants that fitted best with their phenotypic criteria, but have also selected for yield—the seed production of the group (Donald, 1968). The most productive fields may thus have contributed the most to the next generation, leading to selection among fields, a necessary but insufficient condition for KS to occur. Indeed, high genetic relatedness among interacting plants is another necessary condition for cooperation to evolve (Biernaskie, 2022; Hamilton, 1964; Montazeaud, Rousset, et al., 2020).

Promoting high relatedness requires dedicated selection schemes that are unlikely to have been mobilized before the onset of pedigree selection at the end of the 19th century (Allard, 1960; Gayon & Zallen, 1998; Hamilton, 1964; Montazeaud, Rousset, et al., 2020). The pedigree method operates first at the individual‐level and subsequently on single‐plant progenies, and is thus characterized by a shift from individual‐level to group‐level selection accompanied by an increase in relatedness over generations (Murphy, Swanton, et al., 2017; Murphy, Van Acker, et al., 2017). This selection method might have contributed to trait‐blind selection for cooperation (Gayon & Zallen, 1998). Potential examples include decreased leaf area in Pima cotton (Lu & Zeiger, 1994) and more erect leaves in maize (Duvick & Cassman, 1999), two phenotypes associated with reduced competitiveness (Anten & Vermeulen, 2016) and have likely emerged as a by‐product of selection for yield. Likewise, smaller root systems with fewer roots per plant and shorter roots have been observed in modern varieties compared to older forms (Figure 2; Fradgley et al., 2020, but see Gioia et al., 2015). This can be viewed as a reduced investment in resource‐harvesting organs, compatible with indirect selection for increased group performance. Human selection has also promoted cooperation in crops by directly targeting cooperative phenotypes. This was the cornerstone of the “weak competitor” crop ideotype assumed to achieve high yield in high‐planting densities (Biernaskie, 2022; Donald, 1968). In agreement with Donald's idea, the introduction of dwarfing genes to reduce lodging in high inputs (nitrogen, weed, and pathogen controls) supply conditions induced a spectacular yield improvement in wheat and rice (Hedden, 2003).

Cooperation arising from kin selection can be facilitated by the existence of kin recognition that allows cooperative behaviors to be directed preferentially toward kin (Hamilton, 1964; Lehmann & Perrin, 2002). Kin recognition implies that plants display phenotypic plasticity toward reduced competition for resources when growing with kin, leading to increased fitness in kin groups. Preferential helping to relatives might hamper the efficiency of varietal mixtures (Fréville et al., 2019), an advocated practice for mobilizing mechanisms such as niche partitioning and facilitation that may drive positive biodiversity effects on productivity. Convincing evidence of kin recognition is still rare in wild species (Karban et al., 2013; Pennisi, 2019) and in crops. Although crops can indeed display phenotypic plasticity in response to relatedness in cultivated plants (Fang et al., 2013; Murphy, Swanton, et al., 2017; Murphy, Van Acker, et al., 2017; Zhang, Zhang, et al., 2016; Zhang, Liu, et al., 2016), how such plastic response affects fitness remains to be more thoroughly explored, by paying special attention to other confounding effects such as those arising from differences in competitive ability among genotypes (Fréville et al., 2019; Masclaux et al., 2010). Assessing whether interactions mediated by kin recognition might have shifted during domestication and breeding needs further work. Indeed, we first need to test for the existence of kin recognition both in crops and their wild relatives. Then, whenever such kin recognition mechanism does exist, we need to assess whether genetic variation at kin recognition loci might have shifted during crop evolutionary history.

## 6 CONCLUSION, CHALLENGES, AND FUTURE DIRECTIONS FOR PROMOTING BENEFICIAL INTERACTIONS IN AGRICULTURE

Modern agriculture faces the need to maintain high quantity and quality production in a context of increasing food demand, while reducing the environmental costs due to massive use of chemical inputs (Altieri, 1989; Tilman et al., 2011). Promoting beneficial interactions within and between species is a promising avenue to address those challenges, by taking better advantage of biological and ecological processes occurring in agroecosystems. In this review, we showed that beneficial interactions arising from niche partitioning and facilitation have been reduced during crop evolution and breeding. Such trend likely results from a combination of multiple factors, such as the loss of functional diversity and plasticity through genetic bottlenecks and selection, and a relaxation of selective pressures on nutrient‐mobilizing traits through increased chemical inputs. In contrast, beneficial interactions arising from kin selection have likely been promoted in the recent crop evolutionary history since the advent of pedigree selection in the late 19th century, and even more recently since the Green Revolution for direct targeting of cooperative phenotypes. We summarize below and in Table 1 future research directions to help promoting beneficial interactions to meet the challenge of more sustainable agriculture.

**TABLE 1:** Future research directions to promote beneficial interactions in agroecosystems

Source of benefits	Action/Research needed to promote beneficial interaction
Niche partitioning	Identify the traits and genes involved in resource‐use or resource‐acquisition Conduct diversifying selection on these traits and genes to promote niche partitioning between genotypes/species Conduct the selection directly in mixtures instead of monocultures Investigate further the role of trait plasticity in promoting phenotypic divergence and niche complementarity Look for unexploited variation in secondary gene pools (landraces, early domesticated forms, wild relatives)
Facilitation	Better characterize the facilitative potential of known traits such as hydraulic lift, root exudates, phytosiderophore production, etc Identify other traits and genes involved in facilitative interactions Conduct directional selection on these traits to create facilitative varieties/species Look for unexploited variation in secondary gene pools (landraces, early domesticated forms, wild relatives)
Microorganism‐triggered facilitation	Select for genotypes that invest into symbiotic associations (rhizobia or AMF) Select for genotypes able to differentiate among sheeters and true mutualists Select for root morphologies favorable to microorganism symbiosis (e.g., thin roots) Select for genotypes that invest in the CMN Look for unexploited variation in secondary gene pools (landraces, early domesticated forms, wild relatives)
Kin selection	Identify the traits and genes involved in a trade‐off between individual competitiveness and group performance Conduct a directional selection on these traits to target cooperative phenotypes that favor group performance Select directly on group performance early in the pedigree selection schemes Investigate the existence of kin recognition in cultivated species and their wild relatives Look for unexploited variation in secondary gene pools (landraces, early domesticated forms, wild relatives)

Promoting beneficial interactions in agrosystems will be facilitated by the identification of the traits that underlie them. This has been a central focus in ecology (Navas & Violle, 2009; Violle et al., 2009), and has also become a major issue in agronomy since breeding has aimed at reducing intraspecific competition to increase crop yield (Donald, 1968). Still, how to promote beneficial interactions within and between species based on plant phenotypes remains very challenging.

First, we still know very little about both above and belowground traits involved in beneficial interactions within and among species. In particular, root traits that have been largely neglected in breeding programs might offer new opportunities to develop more beneficial crops.

Second, mechanisms underlying beneficial interactions are likely to be dependent on environmental conditions. Indeed, we might expect niche partitioning and facilitation to play a stronger role in more limiting conditions, as described in the framework of the Stress Gradient Hypothesis—SGH (Maestre et al., 2009). At the intraspecific level, the recent meta‐analysis of Reiss and Drinkwater (2018) showed that the yield benefit of mixing cultivars was strong under low levels of soil organic matter and nutrient availability. At the interspecific level, intercropping of cereal and legume species improve soil phosphorus use efficiency, especially at low soil P levels (Betencourt et al., 2012; Darch et al., 2018). Likewise, a compilation of 29 studies indicates that grain–legume and cereal intercropping enhances nitrogen use efficiency by stimulating N_2_ fixation in the former and soil N acquisition in the latter, a complementarity effect that disappears with the application of N fertilizers (Rodriguez et al., 2020). We thus expect favorable trait combinations to differ depending on environmental conditions. Linking agronomy and ecology could thus help identifying relevant trait combinations that promote beneficial interactions in low‐input agriculture.

Third, traits involved in plant‐resource acquisition, and therefore potentially important for complementarity and facilitation, can display trade‐offs between them. For example, root diameter correlates positively with the amounts of carboxylates and phosphatase activity in the rhizosheath as well as with AMF colonization across 16 crop species in limiting soil P (Wen et al., 2019). Describing those tradeoffs could help guiding species and variety mixtures, and better predicting correlated selection responses when targeting interesting phenotypes in plant breeding programs.

Fourth, our current understanding on plant–plant interactions mediated by microorganisms is still very limited. A recent study suggests that maize plants trigger AM fungal colonization in mutant neighboring plants displaying deficiency in the mycorrhizal Pi uptake pathway, through nutrient delivery to the CMN (Fabianska et al., 2020). This example opens interesting perspectives for the selection of genotypes that invest in the CMN.

Finally, it is likely that favorable trait combinations will differ depending on the objectives targeted by farmers. For instance, the traits affecting durum wheat mixture performance were different when considering yield or grain quality (Montazeaud, Violle, et al., 2020). Similarly, the favorable trait combinations in species mixtures depend on the targeted objectives for each species (Haug et al., 2021).

Niche partitioning, facilitation, and kin selection all depend on genetic similarity among interacting plants and can thus be promoted by manipulating genetic diversity. Yet, whereas niche complementarity and facilitation are promoted by genetic dissimilarity among plants, kin selection relies on high genetic similarity among interacting plants and often leads to monomorphic stands with only one cooperative phenotype maximizing group productivity (Montazeaud, Rousset, et al., 2020). There is thus a need to jointly mobilize multiple mechanisms by growing plant communities with high phenotypic variability for traits triggering niche complementarity and facilitation, and low variability for traits which for kin selection promotes only one cooperative phenotype.

Such idea fits with the many ecological and agronomic studies showing that phenotypic diversity can have both positive and negative effects depending on the trait. For instance, the relative performance of durum wheat genotype mixtures was positively impacted by between‐genotypes differences in seminal root branching intensity, but negatively impacted by differences in tiller number (Montazeaud, Violle, et al., 2020). Similarly, recent studies suggest that multiple mechanisms can jointly be at play. For instance, root exudates that trigger plastic responses to kin also trigger plant‐microbial symbiotic associations and affect interactions between plant species (Xu et al., 2021). Results in the Douglas fir indicate that carbon transfer via ectomycorrhizal fungi could be significantly greater among kin than nonkin pairs as a result of greater inter‐root fungi biomass and/or increased inter‐root signaling among kin (Pickles et al., 2017). Along this line, Anten and Chen (2021) have proposed the interesting idea of kin recognition offering an added selective advantage for investing into a CMN shared with kin. Overall, these results call for solving the equation of how to combine favorable trait values of crops for boosting beneficial intra‐ and interspecific interactions by promoting phenotypic diversity or phenotypic uniformity depending on the trait (Barot et al., 2017; Haug et al., 2021; Litrico & Violle, 2015; Montazeaud, Rousset, et al., 2020; Montazeaud, Violle, et al., 2020).

Promoting beneficial interactions arising from niche partitioning and facilitation within and between species calls for extending the genetic and functional diversity currently used in agrosystems. The evolutionary history of crops has been characterized by recurrent bottlenecks and directional selection on genes that determine desirable phenotypes, leading to massive loss of genetic diversity (Gaut et al., 2018). As a result of genetic diversity loss, the diversity of traits related to resource acquisition and use that can be mobilized to promote niche partitioning and facilitation has also been reduced, either directly because they were targeted by selection, or indirectly as a result of loss of adaptive genetic diversity accompanying domestication and breeding. There is a clear need to both reintroduce functional diversity from wild gene pools for traits related to resource acquisition and use, and to select genotypes better able to recruit soil compounds in low‐input settings. In addition, sustainability would greatly benefit from breeding for genetic factors involved in plant interactions with microorganisms, such as nodulation capacities, root exudates (Preece & Penuelas, 2020), carbon delivery to fungi, and those impacting the cost‐benefit balance of AMF colonization (Sawers et al., 2008). This could include the engineering of symbiosis pathways by transferring specific genetic innovations from distant plant lineages to improve crops (Delaux & Schornack, 2021).

An alternative approach to promote beneficial interactions is the use of trait‐blind approach. This has been the rationale of the “mixing ability” approach, where mixing ability of the mixture components are estimated based on their observed performances, and used to assemble relevant components (see Barot et al. (2017) for additional details, Forst et al. (2019) for an application in wheat, and Haug et al. (2021) for mixtures of barley and pea). This is also the rationale of trait‐blind selection schemes that create the conditions for cooperation to evolve as a result of kin selection (Biernaskie, 2022; Montazeaud, Rousset, et al., 2020). Promoting cooperative phenotypes by kin selection can be achieved by strengthening the degree of among‐groups selection in selection schemes (Biernaskie, 2022; Montazeaud, Rousset, et al., 2020; Murphy, Swanton, et al., 2017; Murphy, Van Acker, et al., 2017). Current pedigree selection relies on selection at the individual level in the first generations and could thus be even more efficient in improving yield by selecting at the group level from the very beginning. Such selection schemes have proved very efficient in poultry breeding to increase egg production in multiple‐bird cages while reducing aggressive behaviors among individuals (Muir, 1996, 2005). Trait‐blind approaches have also been developed at the genomic level to investigate how allelic and genotypic diversity affects crop performance under different environmental conditions (Subrahmaniam et al., 2018). For instance, allelic diversity at specific DNA regions in experimental stands of *Arabidopsis thaliana* has been shown to promote stand‐level productivity, through an effect on plant‐soil interactions (Wuest & Niklaus, 2018) and flowering time (Turner et al., 2020). Although very promising in the context of mixtures, such genomic approaches have only been recently applied in crops (Montazeaud et al., 2022).

## CONFLICT OF INTEREST

The authors declare no conflict of interest.
